# Supplementary material for: Are carbon emissions trading and green financial instruments synergistic? -Comprehensive quantitative research based on content analysis
Source: PLoS One. 2024 Mar 7;19(3):e0298601. doi: 10.1371/journal.pone.0298601 (PMC10919601; doi:10.1371/journal.pone.0298601)
Supplement: S1 Data — (ZIP) [file pone.0298601.s001.zip › Data/地级市Z_F工作报告.zip!/╣≤╓▌/░▓╦│╩╨/└┤╫╘╣1⁄2╓┌║┼í╛┬φ┐╦╩2╛▌═°í┐_2007.docx]

各位代表：

现在，我代表市人民政府向大会作工作报告，请予审议，并请各位政协委员提出意见。

过去六年工作的回顾

撤地设市以来的六年，市人民政府在市委的领导下，在市人大和市政协的监督、支持下，坚持以邓小平理论和“三个代表”重要思想为指导，全面贯彻落实科学发展观，抢抓西部大开发、新阶段扶贫开发和安顺作为贵州省优先发展重点旅游区的机遇，深化市情认识，不断丰富和完善发展思路，大力实施农业稳市、旅游兴市、环境立市、工业强市战略和人才战略，锐意进取，勇于克服前进道路上的各种困难，胜利完成了市一届人大一次会议确定的目标任务，经济持续快速协调健康发展，社会事业全面进步，人民生活水平明显提高，社会主义民主法制和精神文明建设取得新成绩。

一、经济结构调整迈出新步伐，国民经济保持良好发展势头

综合实力上了新台阶。预计全市生产总值由2000年的60.50亿元提高到2006年的120亿元以上，年均增长 11.5%，人均生产总值由2431元提高到4540元，年均增长10.4%。预计财政总收入、地方财政收入分别由6.27亿元、3.64亿元提高到18亿元、10.50亿元，年均分别增长19.2%、19.0%，到2005年财政总收入由全省倒数第一位上升到第七位，人均地方财政收入由全省第六位上升到第三位。预计社会消费品零售总额由15.97亿元提高到33.48亿元，年均增长13.2%。三次产业排序由二、三、一调整为三、二、一，产业结构进一步优化。

“三农”工作进一步加强。认真抓好农业综合开发、坡改梯、水利、农村电网改造等工程的实施，加大科技兴农力度，粮食产量稳定在76万吨左右。油菜、蔬菜、烤烟、茶叶、山药、中药材等优势农产品产量和效益不断提高，“一乡一特、一村一品”成效明显。畜牧业进一步发展，建立了良种繁育、疫病防治和饲草饲料生产体系，今年畜牧业预计实现产值15.1亿元，占农业产值的比重提高到38.5%。积极推进农业产业化，省级龙头企业发展到5家，市级龙头企业发展到16家，有力地带动了农村经济的发展。新阶段扶贫开发取得阶段性成果，共投入扶贫资金20.28亿元，实施扶贫项目4778个，减少农村贫困人口3.09万人，实现贫困人口易地搬迁1.87万人。深入实施“四个一”工程，开展各类培训183万人次。启动了新农村建设试点规划编制工作，新农村建设开局良好。

工业经济结构优化。预计工业增加值由16.74亿元提高到36.81亿元，年均增长10.9%。全市形成了以能源、机械、化工、医药、食品为骨干的工业体系。相继建成了安顺电厂二期、引子渡电站、500千伏输变电工程和500千伏换流站，新增装机96万千瓦，安顺成为“西电东送”的重要基地。建立与军工企业的协调联系制度，着力为军工企业创造良好的发展环境，完成了部分军工企业周边环境整治规划编制工作，军工企业军品、民品生产全面发展，以军工企业为主的机械制造业整体水平有较大提升。红星发展、南风日化等企业实力进一步增强，化工产业不断发展壮大。百灵制药、牛来香成长为全国知名企业，制药和食品加工业成为富有发展潜力的新兴产业。

旅游业发展加快。安顺成功申报为贵州省优先发展重点旅游区。进一步理顺了旅游管理体制，实现了旅游秩序、经营管理的全面好转。旅游基础设施进一步完善，综合服务接待能力提高。黄果树、龙宫、九龙山国家森林公园、屯堡文化旅游区等景区深度开发加快。关岭古生物化石群国家地质公园和夜郎洞、神龙洞等新的景区景点向游客开放。黄果树景区和屯堡文化列入国家自然和文化遗产名录。格凸河成功申报为国家级重点风景名胜区，夜郎湖、花江大峡谷、天台山—斯拉河成功申报为省级风景名胜区。连续举办了三届黄果树瀑布节和四届油菜花节，黄果树瀑布节成为全国知名的重要旅游节庆品牌，极大地提高了安顺的知名度和美誉度。红色旅游、乡村旅游、工业旅游等各类旅游持续升温。加大旅游宣传促销力度，多次组织开展了国内外大型宣传推介活动，游客逐年大幅度增长。预计今年接待游客750万人次、旅游收入40亿元左右，分别增长4倍、12倍，旅游业成为全市发展最富活力的新兴产业和最具潜力的经济增长点。

二、基础设施建设加快，城市形象和功能明显提升

基础设施建设得到加强。预计全社会固定资产投资累计完成290亿元，年均增长17.1%；其中纳入地方统计的完成156.14亿元，年均增长15.3%。以公路为重点的交通基础设施建设进一步加强。清黄高速公路和关兴、水黄高等级公路建成通车。建设县乡公路626公里，新建和改扩建农村等级公路1314公里、通村公路4235公里。实现了乡乡通公路，乡镇通油路率达71%，行政村通公路率达69%。株六铁路复线建成开通，黄织铁路开工建设。黄果树机场建成通航。形成了公路、铁路、航空三位一体的交通网络。以解决工程性缺水为重点的水利设施建设取得新成绩。实施了黄龙湖（王二河水库）、虹山水库除险加固、安西灌区等重点水利工程，建成“三小”、“渴望”、“解困”等工程7624处，新增有效灌溉面积12.11万亩，解决了52.14万人和14.26万头大牲畜的饮水困难。光照电站建设顺利推进。黔中水利枢纽工程、黄家湾水利枢纽工程和董箐、马马崖电站前期工作进展顺利。以退耕还林为主的生态建设进一步加强。完成营造林153万亩，森林覆盖率达到32%。建设农村沼气池5.35万口，治理水土流失面积463.52平方公里。开展了石漠化治理试点工作。

城乡面貌进一步改善。编制完成了《安顺市城市总体规划》、《安顺市市域城镇体系规划》等一批规划，今年市级详规覆盖率达95%以上，各县区详规覆盖率平均达71%。完成了南华路、塔山东西路、安顺大桥、龙泉路、塔山公园、若飞广场、文庙环境整治、贯城河治理一期和市垃圾无害化处理项目等工程，启动了中华东西路改扩建、虹山环湖路、贯城河治理二期和市污水处理项目等工程。实施“畅通工程”，深入开展“卫生与秩序”等创建活动，完成了城市地名命名工作。安顺市被评为省级历史文化名城。城市建成区面积进一步扩大，城市化率达30%，以城市为中心组织区域经济社会发展的格局初步形成。各县城建设改造力度加大，相继完成了自来水管网改造等一批基础设施项目，环境得到改善，促进了县域经济发展。认真抓好小城镇的规划建设，各乡镇基础设施不断完善，对产业发展的支撑作用进一步增强。

三、着力创新体制机制，改革开放取得突破性进展

各项改革全面推进。深化安顺多种经济成分共生繁荣试验区改革，全面抓好把试验区改革扩大到全市各项工作的落实。大力支持鼓励和引导非公有制经济发展，2005年非公有制经济实现增加值35.7亿元，占全市GDP的33.66%。全面落实“两减免、三补贴”等支农惠农政策，农村综合配套改革稳步实施。粮食流通体制改革顺利推进。市、县区属国有企业改革改制工作力度加大，共完成136户企业的改制工作，占总数的70%。组建了单一法人社性质的城市信用社，并积极推进城市信用社向城市商业银行方向发展。深化投资管理体制改革，拓宽投融资渠道，与国家开发银行开展了“政银合作”。组建了安顺市城市建设投资公司、黄果树景区建设投资公司。深化财政管理体制改革，实行部门预算、国库集中收付、政府采购和“收支两条线”等制度，在全省率先实行会计集中核算制度。深化土地管理制度改革，国有经营性土地出让一律实行招拍挂。认真实施公务员法，人事制度改革加快。建立激励机制，实施建设经济发展先进县区工作，推进经济强县建设。

对外开放势头良好。深入开展“建设诚信安顺、优化发展环境”、“群众评议机关”、“行风评议”和走访百家企业等活动，促进了政风、行风和机关作风的转变。创新招商引资机制，制定了招商引资奖励暂行办法、招商引资鼓励办法、委托招商暂行办法和进一步优化发展环境的系列配套政策措施，市和部分县区成立了政务服务中心，实行“一站式”服务，预计六年来共引进资金55.45亿元。开展多种形式的对外交流，加强了与“9+2”泛珠三角经济区等区域的联系与合作，积极参与贵阳城市经济圈建设，与青岛市的对口帮扶工作进一步加强，与贵州大学开展了市校合作，与日本那智胜浦町结成友好瀑布关系。

四、经济社会协调发展，人民生活水平跃上新台阶

各项社会事业取得新成绩。全市整体通过省“两基”验收，“普九”人口覆盖率由19.63%提高到100%，青壮年非文盲率达到98.67%。完善义务教育投入保障机制，农村义务教育全面实行了“两免一补”。新建和改造中小学学校511所。组建了安顺职业技术学院和安顺学院。评选和实施了一批市级科技创新成果，评选了市级专家，对高级专家和高学历人员实行了津贴补助。深化新闻、文化体制改革，形成了“两报、两台、三中心、一团”的发展格局。建成一批基层文化活动场所。大力实施“村村通”、“远教”和信息共享工程，广播和电视覆盖率分别达到86%和91%。数字电视建设工作加快推进。继承创新黔中文化，熔铸安顺人文精神，实施文化精品战略，创作了一批富有地方特色的优秀文艺作品。安顺地戏、铜鼓十二调列入国家第一批非物质文化遗产名录。建成了市紧急救援中心和市县疾控中心、传染病院（区），新建（改扩建）了一批乡镇卫生院和村卫生室，初步建立起了疾病预防控制体系、卫生监督体系和医疗救治体系。实行乡镇卫生院财政全额预算管理。新型农村合作医疗试点参合率达72.8%。创新人口计生工作机制，实施“少生快富”工程，全面推行人口计生工作村（居）民自治，全市实现了向低生育水平的转变。人口出生率和自然增长率分别由19.9‰、13.1‰下降到13.2‰、7.1‰。全民健身运动广泛开展，竞技体育水平提高。全面落实民族宗教政策，民族地区经济社会持续发展，宗教工作有序推进。老干、档案、史志、外事、侨务、对台等工作进一步发展。

城乡居民得到更多实惠。坚持实施积极的就业政策，全面促进就业和再就业。累计新增就业7.74万人，其中下岗失业人员再就业1.56万人（“4050”人员再就业1372人），城镇登记失业率控制在4.4%以内。农民人均纯收入、城镇居民人均可支配收入分别增加468元、2704元。全面实行了基本养老、基本医疗、失业、工伤和生育保险，社会保险体系框架基本建立，保障水平进一步提高。“三条保障线”衔接良好，城市低保实现应保尽保，建立了农村特困群众救助制度。不断加大环境保护和污染治理力度，环境质量总体趋于良好。坚持每年集中办一批与人民群众切身利益密切相关的实事。第一期经济适用住房和廉租房建成并向城市中低收入者出售和出租。

五、民主法制和精神文明建设进一步加强，社会稳定工作成效显著

民主法制和精神文明建设不断推进。自觉接受人大的法律监督和工作监督，自觉接受政协的民主监督。认真办理人大代表的建议和政协委员的提案，共办理建议459件、提案1205件，做到了件件有答复，事事有回音。认真听取各民主党派、工商联和人民团体提出的意见和建议，积极支持工会、共青团、妇联、科协、残联、红十字会等人民团体依法按章履行职责。自觉接受新闻舆论监督，重视人民群众对政府机关及其工作人员的监督。政务公开、厂务公开、村务公开力度进一步加大。大力实施“四有”工程，农村基层组织建设进一步加强，村（居）民自治工作深入推进。扎实开展“四五”普法教育，干部群众的法治观念进一步增强。贯彻落实《公民道德建设实施纲要》，加强社会公德、职业道德和家庭美德教育，全民思想道德素质不断提高。积极推进“八荣八耻”社会主义荣辱观实践活动。大力加强和改进未成年人思想道德建设和大学生思想政治教育。推进国防建设与经济建设协调发展，“双拥”工作得到加强。“整脏治乱”专项行动初见成效，“三下乡”、“四在农家”等活动深入推进，广泛开展“文明单位”、“文明村寨”、“文明机关”等创建活动，涌现出一批全国、全省文明单位和文明村寨，全社会的文明水平不断提高。

切实维护社会稳定。制定了《安顺市重大突发公共事件总体应急预案》及37个子预案，组建市应急办，建立了市级重大突发公共事件应急管理平台。切实搞好社会治安综合治理，开展“平安安顺”创建活动，严厉打击“法轮功”等邪教组织和刑事犯罪，确保社会政治稳定。建成了一批基层司法所、派出所和法庭。贯彻落实《信访条例》，坚持和完善领导干部信访接待日制度，重视解决群众的合理诉求，认真做好征地、拆迁、移民、国有企业改制等方面涉及群众切身利益的工作。注意发挥人民调解、司法调解在化解人民内部矛盾方面的作用。严格落实安全生产责任制，安全生产工作不断加强。

六、政府职能转变加快，工作效率进一步提高

坚持精简、统一、效能的原则，进一步转变政府职能。在继续加强经济调节和市场监管的同时，更加注重社会管理和公共服务职能。贯彻落实《行政许可法》和《国务院依法行政实施纲要》等法律法规，创新行政管理方式，民主决策、科学决策和依法行政的能力和水平不断提高。行政审批制度改革取得阶段性成果，全市取消行政许可193项。完成了市、县区政府机构改革。制定了《安顺市人民政府工作规则》，提高了政府及政府部门工作的规范化和制度化水平。进一步拓宽群众对行政事务知情、参与和监督的渠道，不断提高政府公信力。推进电子政务建设，开通了安顺市人民政府网站。加大反腐倡廉力度，努力解决损害群众利益的突出问题。加强对重点环节和重点部位权力行使的制约和监督，政府投资项目全面实行了招投标制和代建制，治理商业贿赂专项工作扎实推进。政府系统干部队伍建设加强，素质提高。

各位代表，过去的六年我们在各个方面取得了明显的成绩，今年经济社会发展的各项目标任务也将如期全面完成和超额完成。预计全市生产总值120亿元以上，同比增长14%以上；财政总收入18亿元，同比增长22.4%，其中地方财政收入10.50亿元，同比增长26.2%；全社会固定资产投资完成55.57亿元，同比增长18%，其中纳入地方统计的完成38.12亿元，同比增长18%；农民人均纯收入1940元，同比增长6%；城镇居民人均可支配收入8580元，同比增长15%。粮食产量77.84万吨，创历史最高水平；国有企业改革改制工作加快推进，安顺试验区申报国家综合配套改革试点工作进展顺利；成功举办了2006中国·贵州黄果树瀑布节和首届贵州旅游产业发展大会，“三赛一会”活动成效明显；顺利完成了以黄果树大街改造一期为代表的城市建设和以黄果树新城为代表的旅游基础设施建设“8·31”工程，创造了“安顺速度”，展示出安顺干部群众新的精神风貌，开创了我市提速发展的新局面。各条战线、各个方面的工作呈现出良好态势，实现了“十一五”的良好开局，为奋力提速发展，实现黔中崛起，加快安顺经济社会发展的历史性跨越，建设繁荣富裕秀美文明和谐的新安顺奠定了基础。

过去六年取得的成绩，为我们今后发展积累了许多宝贵的经验：

──坚持以科学发展观统领全局，抓好发展第一要务不放松，大力加强经济建设，统筹推进经济社会协调发展，树立正确的政绩观，一切从市情出发，充分抓住机遇，用好机遇，不争论，不折腾，集中力量谋发展、抓发展。

──坚持以改革促发展，以开放促开发，以创新促跨越，不断深化安顺试验区改革，实行全方位的对内对外开放，坚决破除影响发展的一切观念和做法，努力通过体制机制创新，增强发展的内在活力，通过思想的大解放，促进各项工作的大提高。

──坚持以人为本，关注民生，切实实现好、维护好、发展好广大人民群众的根本利益，认真解决人民群众最关心、最直接、最现实的利益问题，让发展的成果惠及全体人民。

──坚持以结构调整为主线，切实转变发展观念，转变增长方式，提高发展质量，紧紧把握住全市发展的重点领域和关键环节，大力推进产业结构优化升级，不断提高产业竞争力。

──坚持调动一切积极因素，充分发挥各方的积极性、主动性和创造性，形成加快发展的合力，大力支持各县区走符合自身实际的发展道路，不断壮大县域经济。

──坚持把提高执行力摆在政府自身建设的重要位置，健全抓落实的制度，创新抓落实的机制，强化抓落实的纪律，形成各级各部门齐心协力抓落实的良好局面，确保各项工作取得实实在在的效果。

过去六年所取得的成就，是全市各级各部门解放思想、与时俱进、求真务实、开拓进取的结果，是全市各族人民团结奋斗、迎难而上、辛勤劳动的结果，是社会各界通力合作、大力支持的结果。在此，我代表市人民政府，向全市各族人民，向人大代表、政协委员，向各民主党派、工商联、无党派人士、各人民团体，向驻安人民解放军指战员、武警官兵和公安干警，向中央和省在安单位，向所有关心、支持安顺发展的各界人士和海内外朋友们，表示衷心的感谢，并致以崇高的敬意!

在充分肯定成绩的同时，我们也要清醒地看到欠发达和欠开发并存的基本市情仍然没有得到根本性改变，我们在前进中还存在不少困难和问题，主要是：经济总量较小，经济结构有待进一步优化；体制机制性矛盾尚未根本解决，工业化、城市化、市场化水平低；农民增收渠道还不多，新阶段扶贫开发任务仍然十分繁重；提供公共服务的能力不强，一些社会矛盾还比较突出，就业和再就业的压力仍然较大；政府职能和工作作风与社会主义市场经济的要求还有不相适应的地方，执行力需进一步提高，廉政勤政建设有待进一步加强。对于这些问题，我们将高度重视，切实加以解决。

今后五年的奋斗目标和工作任务

今后五年是奋力提速发展，实现黔中崛起，加快安顺经济社会发展的历史性跨越，建设繁荣富裕秀美文明和谐新安顺的关键时期，我们必须充分认识到发展对安顺尤为重要，牢牢把握好当前面临的难得机遇和有利条件，坚持以思想大解放促进经济大发展，把观念更新作为思想解放的切入点，把破解难题作为思想解放的突破口，把振奋精神作为思想解放的着力点，充分调动广大人民群众的积极性、主动性和创造性，为实现市第二次党代会提出的目标任务而不懈努力，勤奋工作。

今后五年政府工作的指导思想是：

坚持以邓小平理论和“三个代表”重要思想为指导，以科学发展观统领经济社会发展全局，深入学习贯彻党的十六大，十六届三中、四中、五中、六中全会精神，省委九届各次全会和市第二次党代会精神，全面加强政府自身建设，努力开创社会主义经济建设、政治建设、文化建设和社会建设的新局面，奋力提速发展，实现黔中崛起，加快安顺经济社会发展的历史性跨越，建设繁荣富裕秀美文明和谐的新安顺，为全面建设小康社会奠定坚实基础。

到2011年的目标任务是：

──建设经济繁荣的新安顺。国民经济实现提速发展，综合实力和竞争力不断增强。全市生产总值达到280亿元左右，人均达到1200美元左右；财政总收入达到38亿元左右，其中地方财政收入达到22亿元左右。

──建设人民富裕的新安顺。人民群众生活更加宽裕，家庭财产普遍增加，收入水平和生活质量明显提高，公共服务设施日臻完善。城镇居民人均可支配收入达到12000元左右，农民人均纯收入达到3000元左右，城乡居民人均居住面积达到25平方米以上。

──建设山川秀美的新安顺。生态环境进一步优化，可持续发展能力明显增强。万元生产总值综合能耗明显下降，全市森林覆盖率达到40%以上，人口自然增长率低于全省平均水平；城市建成区绿化覆盖率达到35%以上，城镇污水处理率和城市生活垃圾无害化处理率达到60%以上，环境空气质量优良率达到95%，城市燃气化率达到50%以上。

──建设城乡文明的新安顺。“三个文明”建设协调发展，全社会文明程度进一步提高。城市的要素集聚能力、基础设施承载能力和辐射、带动、服务功能显著增强。全市城市化率达到35%以上。社会主义新农村建设取得明显成效。

──建设社会和谐的新安顺。社会建设与经济建设、政治建设、文化建设协调发展，人民群众安居乐业。社会主义民主得到充分发扬，各方面的积极因素得到广泛调动，社会各方面利益关系得到妥善协调，劳动就业、社会保障基本政策得到全面贯彻，各项社会事业全面发展。形成民主法治、公平正义、诚信友爱、充满活力、安定有序、人与自然和谐相处的基本社会格局。

为实现上述目标，我们将切实抓好以下工作。

一、抓住机遇，夯实新安顺产业基础

深入实施农业稳市、旅游兴市、环境立市、工业强市战略，全面实施人才战略，抓住机遇、用好机遇，坚持用发展的办法解决前进中的问题，努力实现农业、工业和以旅游业为龙头的第三产业又好又快发展，尽快改变我市欠发达、欠开发的状况。

（一）发展农业农村经济，扎实推进社会主义新农村建设

坚持多予少取放活的方针，按照“生产发展、生活宽裕、乡风文明、村容整洁、管理民主”的要求，以富裕农民为中心，以发展现代农业为支撑，以加强公共设施建设为突破口，以提高农民素质为关键，全面推进社会主义新农村建设，努力实现农村经济有新发展，农民生活水平有新提高，农村面貌有新改善。

大力调整农业产业结构。坚持走内涵式发展粮食生产的路子，实施边远地区粮食增产工程、大田大坝“双增”工程、公路沿线农业示范工程，确保粮食年均产量不低于76万吨。进一步建立健全畜禽良种繁育、疫病防治、饲草饲料生产、质量安全和畜产品市场体系，抓好小区养殖和规模养殖，力争到2011年全市畜牧业产值达到22亿元，占农业总产值的比重提高到45%以上。在大力实施“一乡一特、一村一品”的基础上，做大做强特色产业，建设好优质米、油菜、蔬菜、烤烟、茶叶、中药材6大产业基地。用工业理念经营农业，大力推进农业产业化。建立龙头企业发展基金，积极培育发展龙头企业，引进一批国际国内优强企业，形成一批产、供、销一体化的龙头企业。申报一批国家级、省级重点龙头企业，市级重点龙头企业达到25个，龙头企业加工原料来自市内基地的比重达到80%以上，农民人均纯收入中有35%左右来自农业产业化经营。

着力改善农村生产生活条件。切实加强农村水、电、路、能源、通信等基础设施建设。保障农民土地承包经营的各项权利，实行最严格的耕地保护制度。加大基本农田保护和建设力度，实施“沃土工程”，完成中低产田土改造20万亩。以解决工程性缺水为目标，争取开工建设黔中水利枢纽工程，抓好“滋黔工程”的实施，搞好黄龙湖灌溉渠系配套工程，大力实施雨水积蓄利用“益民工程”，高质量抓好烟水配套工程建设，不断提高水资源的调配能力。到2011年，全市新增有效灌溉面积31万亩，力争建成人均0.5亩基本农田，解决30万人饮水安全问题。加强新农村建设规划工作，为全市乡村建设提供规划服务。搞好山水林田路综合开发，大力实施沼气工程和“改厨、改厕、改圈”工程，每年新建沼气池2万口以上。

建立健全农村社会化服务体系。加快农业科技进步，加强农业科技人才队伍建设。大力实施“农业科技入户工程”，努力在推广应用组织培养技术、旱地育种、推广生物多样性栽培、测土配方施肥、推广农业循环经济生产5个方面有新的突破，切实提高农业资源综合利用水平和农业科技水平。完善动植物病虫害防控和气象防灾减灾体系，提高为农服务水平。建立健全农产品质量安全体系、检验检测体系和农业标准化体系，积极发展无公害农产品、绿色食品和有机农产品，种植基地全部实现产地无公害认定。积极发展农村各类专业协会，建立起基本覆盖县区、乡镇、村的各种农村专业合作经济组织，增强农村集体经济组织服务功能，提高农民进入市场的组织化程度。健全农产品市场体系，加快农业信息网络建设，促进农产品流通。做好农业生产资料供应和市场管理。积极推进农业机械化，建设20个农业机械化示范乡镇。深入实施“四有”工程和“四个一”工程，切实加强对农民的培训，造就千千万万有文化、懂技术、会经营的新型农民。

加大新阶段扶贫开发力度。坚持分类指导、整体推进，采取项目带动、易地搬迁、长期救助等扶贫方式，切实搞好整村推进、产业化扶贫、劳动力转移培训三项重点工作。完成991个贫困村的整村推进工作，实施贫困地区劳动力转移培训4万人，搞好易地扶贫搬迁，抓好移民后期扶持工作，基本解决现有贫困人口温饱问题。帮助低收入人口稳定提高生活水平，努力抑制因灾、因病返贫。加大财政扶贫资金投入力度，争取信贷扶贫资金，整合用好各类涉农资金。继续实行定点帮扶、对口帮扶，引导动员全社会力量参与扶贫开发，帮助贫困群众进一步改善基本生产生活条件、拓宽基本增收门路、提高基本素质，增强贫困地区自我发展能力。

（二）走新型工业化道路，全力加快工业经济发展

增强工业在经济发展中的主导地位，努力实现传统产业向现代产业转变、传统机制向现代机制转变，初步形成以高新技术产业为先导、特色优势产业为支撑、基础支柱产业为依托的工业产业格局。到2011年，全市工业增加值达到100亿元以上。

加快工业园区建设步伐。按照“布局集中、土地集约、产业集聚”的原则，科学制定4个工业园区发展规划。加大政策支持力度，逐步建立多元化的园区投入机制，加快园区基础设施建设。引导投资项目向园区集中，把园区建设成为发展新型工业的集中区、吸引外资的集聚区、体制改革的先导区、军品转民品的导入区和循环经济的示范区。重点建设以机械装备制造业为主的双阳机械工业园区和黎阳高新技术产业园区；以旅游商品、药品、食品、农产品加工等为主的西秀工业园区；以原材料等基础工业为主的普定循环经济工业园区。全力打造2个销售收入上100亿元、2个销售收入上50亿元的工业经济板块，5个以上销售收入上10亿元、20个以上销售收入上亿元的企业，培养新的经济增长极。

优先发展机械装备制造业。发挥军工企业技术、设备及人才优势，支持研发生产科技含量高、市场前景好的民用产品，加快发展民用飞机及航空零配件生产，努力把安顺建成全国飞机及系列产品的研发和制造基地，积极争取将双阳机械工业园区打造成全国知名的航空城。加快汽车整车和零部件企业发展，推进云雀轿车项目资产重组。支持复合材料、铁路机车配件等项目开发。加快发展以装备工业为代表的先进制造业，开发市场潜力大、附加值高的设备和产品，做优做精医疗器械、石油钻井机具、采棉机械、大型成套设备零部件、精密轴承和石材加工机械等产品。鼓励研制开发生产环保、能源、工程、农业和园林机械以及大型成套设备和基础件，形成更多拥有自主知识产权的知名品牌。

加快发展旅游商品和制药等特色轻工业。进一步整合生产要素，大力发展融地方性、民族性、文化性于一体，满足多样化需求的旅游商品。开发具有个性化特点的各景区旅游纪念品。依托品牌产品及骨干企业，大力发展波波糖、牛肉干、山药、辣椒制品、调味品和精制茶等特色旅游食品。大力开发木雕、根雕、地戏脸谱、民族刺绣、古生物化石仿制品等旅游工艺品。振兴白酒、蜡染、布依地毯、三刀等为主的传统轻工业。努力把安顺建设成为全国重要的旅游商品生产基地。以推进中药现代化为重点，发展以苗药为主、地道药材为特色的多种生产剂型中成药。支持制药企业研发科技含量高、附加值高、市场销售前景好的新产品。支持有条件的中药材种植基地达到国家GAP标准。吸引更多企业到安顺落户，形成有竞争力的民族制药企业集群。

优化发展能源等基础工业。积极配合光照、董箐、马马崖水电站建设，开发建设打邦河、清水河、蒙江河流域小水电站。扎实做好煤炭资源勘查及矿区总体规划，加快煤炭生产结构调整，建设一批安全、高效、低排污的大中型骨干矿井，促进煤炭产业从粗放型向集约型转变。大力实施煤电化一体的工业循环经济发展计划，科学整合煤炭开发、火力发电、烟气脱硫及相关产业，使煤电化产业达到效益、环保、节能的集群效应。相对控制重化工产业发展。积极发展风能等新型能源。加快原材料、新型建筑材料的开发和生产。

（三）加快安顺综合旅游区建设，实现旅游产业全面提速

按照“走进安顺、走进自然、走进历史、走进文化”的旅游发展定位，深入贯彻落实省委省政府《关于支持首届贵州旅游产业发展大会承办地安顺市加快旅游业发展的意见》，整合资源，创新发展，提前改善旅游基础设施条件、提前改善生态环境、提前提高旅游接待能力、提前实现旅游业阶段发展目标，把安顺综合旅游区建成在国内外有重大影响的旅游目的地，带动各类产业加快发展。

加强旅游基础设施建设。抓紧编制和完善全市旅游发展总体规划和重点景区详细规划。加快黄果树镇半边街搬迁工作，加大黄果树新城建设力度。建设以九龙山国家森林公园为重点的一批休闲度假村和乡村旅游示范村寨，建设屯堡文化博览园（区）和古生物古人类博物馆。规划建设一批星级宾馆、度假型旅游宾馆和乡村旅馆，到2011年，全市旅游住宿设施床位达到3万张以上，其中星级标准床位达到1.5万张以上。建立健全游客接待服务网络，提高服务水平。进一步加大景区保护力度，抓好景区及其周边的生态环境建设，完成高等级公路10万亩生态景观建设工程。积极开展创建全国优秀旅游城市工作。

大力实施旅游精品战略。推进旅游产品结构调整，在进一步发展自然风光游的基础上，开发丰富多彩的民族风情、历史文化旅游资源，积极发展红色旅游、乡村旅游、休闲度假旅游、科考旅游和工业旅游。精心打造黄果树、龙宫、格凸河和屯堡文化四大旅游品牌，将黄果树、龙宫、格凸河建成一流的喀斯特自然风光旅游和度假精品景区，将屯堡文化旅游区建成一流的历史文化旅游精品景区，将以王若飞故居、安顺府文庙、武庙为重点的历史文化街区建成一流的红色旅游和历史文化旅游精品景区。抓好黄果树景区再次申报世界自然遗产工作，申报花灯、蜡染、屯堡山歌等进入国家第二批非物质文化遗产名录，申报1—2个国家级风景名胜区。建成2个国家5A级旅游景区、3—5个国家4A级旅游景区、2—4个省级风景区，形成一批各具特色的旅游产品。以游客在安顺停留三天以上为目标，优化旅游线路设计，提高游客消费空间和旅游业发展质量。全市接待游客达到1200万人次以上，旅游收入120亿元以上。

深化旅游管理体制改革。按照管住资源、放开经营，控制规划、有序推进，扩大规模、增加收入的要求，深化旅游管理体制改革。加快推进旅游景区所有权和经营权分离，进一步推进风景区项目特许经营，完善旅游企业经营机制。按照现代企业制度的要求，将黄果树旅游集团公司改组为跨区域、跨行业的旅游发展总公司，建成安顺旅游产业的旗舰和对外开放平台，培育成在全省乃至全国有一定影响力和竞争力的大型旅游企业集团，积极创造条件上市融资。组建龙宫旅游开发公司和格凸河旅游开发公司，实现安顺旅游企业集团化、网络化、品牌化。采用合作、股份制等方式进行旅游投资开发，吸纳民间资本发展旅游业。加快旅游人才队伍建设。培育和引进一批大型旅行社，积极申办一批国际旅行社。

大力开拓旅游市场。建立健全全市统一的整体旅游营销体制，全方位、多角度、宽领域，加大旅游宣传推介力度，强化全民服务旅游意识，树立旅游城市新形象。把黄果树瀑布节打造成全国乃至世界知名的旅游节庆品牌，继续办好油菜花节、屯堡文化节。巩固和扩大川、渝、滇、湘和珠三角、长三角、京津及港台地区等重点国内市场，拓展日本、韩国、东南亚、澳洲和欧美市场，努力在入境旅游和高端游客市场上取得突破。加强与周边省区旅游业发展的交流与合作，提高旅游产业竞争力。

大力发展现代服务业。在加快旅游业发展的同时，重视其他产业的发展。加强商业基础设施建设，规划建设一批专业市场，拓展商贸流通业的发展空间，增强商贸流通业的聚集辐射功能，把安顺建设成为区域性商贸中心和物流中心。加快发展房地产市场，进一步搞活房地产二级市场，扩大消费需求。大力扶持特色餐饮、文化娱乐、康体休闲、旅游购物等服务业，积极发展金融、保险、法律、咨询和交通运输等服务业。加强会展场馆建设，大力发展会展经济。大力发展信息产业，加快国民经济和社会信息化进程。

二、打造生态旅游城市，强化新安顺带动功能

按照城市现代化、农村城镇化、城乡一体化的总体思路，加快推进城市化进程，构建百万人口生态旅游城市框架，着力形成“五区三自治县”新的组团式城市发展格局，做大做强做优做美中心城区，提高城市发展水平，充分发挥城市对新型工业化的支撑作用，带动全市经济社会提速发展。

（一）切实加强规划工作

改革城市和乡村分离的规划体系，构建城乡一体的规划新体系。科学确定城市定位，调整优化城市布局，搞好城市形象设计，突出城市个性塑造，丰富城市文化底蕴，体现专业生态旅游城市、国家园林城市、优秀旅游城市和历史文化名城特色，完成城市总体规划和土地利用总体规划修编，为新一轮更大规模的城市建设和城镇化工作的推进奠定良好基础。编制完善县区城镇体系规划和小城镇规划。积极调整优化行政区划，开展撤县设区工作。

（二）加快城市建设步伐

进一步强化经营城市理念，增强城市建设投融资能力。充分发挥基础设施建设引领城市发展的关键性、先导性作用，改善人居环境、完善城市基础设施功能、增强城市可持续发展能力，实现城市面貌一年一变样，五年大变样。每年推出新一轮“8·31”工程。强力推进旧城改造和新城区建设。加快以中心城区二环路工程为代表的城市道路网络建设。完成黄果树大街、中华东西路改扩建和虹山环湖路建设项目。完成中心商业广场、特色文化商业街等建设项目。加快体育中心、虹山公园、青少年活动中心、老年活动中心等场馆建设步伐。完成文庙广场及其周边地段、若飞广场建设控制区的开发建设，加快文物保护单位的修复，实现明清商业街的恢复。完成贯城河二期治理工程，建成市区污水处理厂及收集系统。加快环城林带建设，加大城区周边山头封山育林、植树造林力度，完善中心城区绿化小品和街景的设置，提高城区绿化覆盖率。实现城市公共设施逐渐向周边农村辐射延伸。

（三）加大城市管理力度

建立健全市、区、街道相互衔接、合理分工和规范高效的城市管理框架，完善统一领导、各司其职、规范管理、强化基层的城市管理格局，逐步形成全民参与城市管理的新模式，建立和完善城市管理长效机制。积极推进城市管理相对集中行政处罚权工作。按照投资多元化、管理企业化、发展产业化的思路，加快环卫、园林等行业的改革，逐步形成市政基础设施有偿使用、多渠道养护的管理机制。加强社区管理，完善社区功能，增强社区服务。

（四）加快区域交通网络建设

进一步加强公路、铁路、航空、内河航运等交通基础设施建设，完善综合交通运输体系，建成一小时经济圈。加快建设中心城区和各县区连接的快速交通体系，构建城市道路骨架。继续配合实施镇胜高速公路建设，加快普定—紫云—望谟新公路和龙宫—黄龙湖—黄果树等公路建设步伐，开工建设长顺—紫云—兴仁公路。建设服务于电煤供应、电站建设、工业园区、农业生产基地和重点旅游景区景点的公路，提高公路等级。实现全市100%的乡镇通油路或水泥路，100%的建制村通公路。市内二级以上公路比重提高到20%左右，等外公路比重降至15%以下。配合建成黄织铁路。进一步完善黄果树机场基础设施，开通新的旅游航线。进一步改善内河航运条件，开辟南下珠江的水上通道。

（五）大力推进县城和城镇建设

坚持发展小城镇与壮大区域特色经济、推动农业农村结构调整、优化生产力布局、培育新的经济增长点相结合，加强城镇水、电、路、通信、绿化等基础设施和服务设施建设，增强城镇的辐射功能、服务功能和集聚功能。把平坝、普定建设成为城市副中心，推动关岭、镇宁、紫云自治县向小城市方向发展。继续抓好小城镇建设试点工作，在交通干线、旅游景区周边重点开发建设一批具有明显特色和可持续发展功能的中心镇，逐步形成等级规模适宜、功能互补、布局合理的黔中城镇群。建立有利于城镇发展的新机制，加快生产要素向城镇集中。引导农村劳动力向中心镇、县城和中心城区有序流动。

三、深化改革扩大开放，增强新安顺内在活力

坚持以解放思想为先导，以改革开放为动力，努力实现体制机制创新，消除影响发展的障碍，不断解放和发展生产力，为全市经济社会加快发展增强新的活力。

（一）深入推进安顺试验区改革

紧紧围绕多种经济成分共生繁荣的主题，继续做好安顺试验区申报国家综合配套改革试点工作，抓住时机，加快节奏，深化改革，大力推进思想观念和体制机制创新。坚持以改革促发展，以完善社会主义市场经济体制为目标，大胆探索实践，增强生产要素聚集力，为经济社会发展不断增添新动力。用改革的办法解决发展中存在的体制性、机制性问题，把加快发展作为深化改革的根本出发点，把发展中遇到的难点作为改革的重点，努力发挥改革对解放和发展生产力的促进作用。建设创新型安顺，增强创新能力，最广泛、最充分地调动一切积极因素，最大限度地激发各方面的创造活力，再创安顺试验区体制机制新优势。

（二）大力发展非公有制经济

认真贯彻落实中央、省和市鼓励支持引导非公有制经济发展的政策措施，进一步为非公有制经济快速发展创造公平的竞争环境和宽松的发展环境。鼓励非公有制资本发展高新技术产业、特色优势产业、金融服务业、市政公用事业，参与农业产业化经营和投资国防科技工业。建立中小企业发展专项资金，支持非公有制企业的发展。支持金融机构加大对非公有制经济的信贷力度。支持具备条件的非公有制企业上市融资和发行债券。到2011年实现非公有制经济占全市生产总值比重提高到60%以上。继续深化市、县区属国有企业改革，积极引进各类战略投资者参与国有企业改革改制。大力发展国有资本、集体资本和非公有资本参股的混合所有制经济。

（三）深化投融资和财税金融体制等改革

改进政府投资管理方式，落实投资审批制、核准制和备案制，引导投资方向，提高投资效益。规范政府投资行为，确立企业的投资主体地位。创新政府建设项目融资方式，加快投融资平台建设。深化与开发银行的政银合作，扩大与其他金融机构的合作。继续推进公共财政体制的健全和完善。深化部门预算、国库集中收付、政府采购和“收支两条线”管理改革。强化预算管理，加强预算外资金的监管。加强国有资产管理，确保国有资产保值增值。推进城市信用社、农村信用社的改革和发展，组建城市商业银行和农村合作银行。继续落实“两减免、三补贴”等支农惠农政策，深化农村综合配套改革。全面推行国有经营性土地和工业用地出让招拍挂制度，推进矿产资源有偿出让制度改革。

（四）进一步优化发展环境

深入开展“建设诚信安顺、优化发展环境”创建活动，努力营造良好的发展环境。进一步简化行政审批程序，完善市级政务服务中心，健全县区政务服务中心，规范和完善“一站式”服务体系。以建立长效机制为根本，加强行政效能建设，继续实行首问负责、限时办结等制度，完善投资环境监测、考核、公示制度，严格执行行政不作为和过错责任追究制，切实解决乱收费、乱罚款、乱摊派、乱检查和门难进、脸难看、话难听、事难办等突出问题。继续在全市开展“群众评议机关”、“行风评议”活动，促进政风行风不断改善。完善现代市场体系，加快资本、土地、技术和劳动力等要素市场发展，建立和完善市场信息发布制度。整顿和规范市场秩序，坚决打击制假售假、商业欺诈和各种侵权行为。

（五）全面扩大开放合作

坚持把扩大开放作为加快发展的重要途径，打破所有制和行政隶属关系的制约，充分释放发展活力。加快“引进来”和“走出去”步伐，充分利用两种资源，开拓两个市场。加大招商引资力度，加强招商引资项目库建设，推出一批发展前景好、产业关联度高、集聚效应明显，对全市经济发展有较大带动作用的项目，吸引国内外大企业、大集团投入资金和技术。认真搞好招商引资项目的后续跟踪服务，提高资金到位率。积极参与贵阳城市经济圈建设，强化安顺作为贵阳城市经济圈副中心的地位，实现与经济圈内其他城市的良性互动、优势互补和产业衔接。加强与发达地区、优强企业、科研院所和重点院校的合作与联系。进一步加强与“9+2”泛珠三角经济区、长三角地区和青岛等地的经贸交流与协作。整合建立驻北京、上海、广州等城市的办事机构。鼓励和支持有条件的企业参与国际国内合作与竞争。

（六）加快县域经济发展

建立健全支持县域经济发展的政策措施和激励机制。落实建设经济强县、建设经济发展先进县区和培育支柱财源、扶持县级财政收入上台阶等政策，进一步壮大县域经济。进一步理顺市与县区的关系，合理调整事权，增强县区统筹能力，增强各县区在项目、资金、信息、物资、技术、人才等方面的聚集能力，增强县域经济发展活力。加强对各县区发展的指导，支持各县区发挥比较优势，根据资源特点，科学制定产业发展规划，优化产业布局，大力发展特色产业，培育产业集群，逐步提高产业集中度，实现协调发展和共同发展。支持西秀区搞好“建强”工作，支持平坝县、普定县进入全省经济强县行列，支持安顺开发区、黄果树风景区在经济社会发展中率先发展，支持镇宁、关岭、紫云三个自治县实现跨越式发展。

四、以人为本，统筹新安顺协调发展

按照构建和谐社会的总要求，以解决人民群众最关心、最直接、最现实的利益为重点，大力提高政府公共服务能力，着力发展社会事业、促进社会公平正义、建设和谐文化、完善社会管理、增强社会创造活力，走共同富裕道路，努力实现人与人、人与社会、人与自然相和谐。

（一）加快发展各项社会事业，不断满足人民群众的需求

优先发展教育事业，促进教育公平。坚持把义务教育工作重点放在农村的方针，落实农村义务教育保障机制，实施农村义务教育阶段薄弱学校改造工程，巩固提高“两基”成果。继续在农村并逐步在城市免除义务教育学杂费，全面落实对家庭经济困难学生免费提供课本和补助寄宿生生活费政策，保障和维护农民工子女平等接受义务教育的权益。大力发展各级各类教育，积极普及十二年制教育，办好安顺学院，建设安顺旅游职业技术学院，在每个县区重点办好一所起骨干示范作用的职教中心（中等职业学校）。深化科技体制改革，发挥科技进步对经济社会发展的支撑引领作用，力争科技对经济的贡献率达到40%以上。加强多种形式的产学研联合，大力扶持科技型中小企业和民营科技企业，全面提高企业技术创新能力。加强知识产权保护，做好科学普及工作。大力实施人才战略，健全科学的人才培养、评价、选拔、任用和激励保障机制，切实抓好政府工作人员、专业技术人才和企业经营管理人才队伍建设，加快各类技术职称评审工作，培养一批高级人才，在全社会形成尊重知识、尊重人才、尊重劳动、尊重创造的良好氛围。

推进文化体制改革，抓好公益性文化事业和经营性文化产业的发展。加快博物馆、图书馆、档案馆、文化馆、乡镇文化站、村文化室的建设，建立覆盖城乡的公共文化服务体系。加快“村村通”广播电视工程建设，完成安顺人民广播电台升级改造项目。加快推进文化信息资源共享、农村现代远程教育、数字电视整体平移等工程，搞好文物保护工作，加强民族民间文化和非物质文化遗产挖掘、抢救、保护和发展。把文化产业与旅游业结合起来，以项目整合资源，带动文化产业发展。健全和完善疾病预防控制、卫生服务、医疗救治、卫生执法监督体系。加强公共医疗卫生设施建设，完成乡镇卫生院的房屋改造和基本设备配置，50%以上的村卫生室达到甲级标准。大力开展新型农村合作医疗，参合率达90%以上。大力发展社区卫生服务。加强食品药品安全质量监管。整顿规范医药市场和医疗收费，努力解决群众看病难看病贵问题。切实做好艾滋病等重大传染性疾病和地方病的防治工作。加强城乡社区体育设施建设，广泛开展全民健身运动，提高竞技体育水平。

（二）高度关注民生，努力提高人民生活水平

积极发展就业容量大的劳动密集型产业、服务业、非公有制经济和中小企业，多渠道、多方式增加就业岗位。落实促进就业的财政金融政策，鼓励自主创业和自谋职业。扩大再就业政策扶持范围，加快再就业援助机制建设，着力帮助零就业家庭和就业困难人员就业。促进城乡统筹就业，进一步拓展农村劳动力就业空间，维护劳动者特别是农民工合法权益，发展和谐劳动关系。加强对劳动者的职业技能培训、创业培训和再就业培训，逐步形成城乡统一的人才市场和劳动力市场。逐步健全社会保险、社会救助、社会福利、慈善事业相衔接的覆盖城乡居民的社会保障体系。多渠道筹集社会保障基金，不断完善社会保障制度，努力扩大参保人口覆盖面。积极探索多种形式的农村养老保险制度和解决失地少地农民、农民工社会保障问题的新路子，逐步建立农村最低生活保障制度。加强对困难群众的救助，完善城市居民最低生活保障、农村五保供养、特困群众救助、灾民救助、城市生活无着的流浪乞讨人员救助等制度。加强经济适用住房和廉租房建设，逐步改善城镇中低收入家庭住房困难问题，提高生活质量。加快农村危房、茅草房改造。多渠道增加城乡居民收入，完善以按劳分配为主体、多种分配方式并存的分配制度，规范收入分配秩序，完善工资正常增长机制。注重社会公平，提高低收入者收入水平，扩大中等收入者比重，调节过高收入，保护合法收入，取缔非法收入。

（三）加强和改善社会管理，切实维护社会稳定

健全党委领导、政府负责、社会协同、公众参与的社会管理格局。充分发挥城乡基层自治组织协调利益、化解矛盾、排忧解难的作用，充分发挥各类社会组织提供服务、反映诉求、规范行为的作用，妥善处理不同利益群体关系。正确处理新形势下的人民内部矛盾，认真做好人民群众来信来访工作，畅通诉求渠道。建立健全社会预警体系和应急救援社会动员机制，提高处置突发事件的能力。完善社会稳定工作责任制，大力加强社会治安防控体系建设，落实综合治理的各项措施，深入开展“平安安顺”创建活动，强化禁毒工作，依法严厉打击“法轮功”等邪教组织和各种犯罪活动。抓好城乡派出所、乡镇司法所和法庭的建设。高度重视隐蔽战线工作，切实维护国家安全。健全统一协调的安全生产监管体制，以煤矿、交通和消防为重点，完善和落实安全生产责任制和安全事故责任追究制，确保安全生产状况进一步好转。加强对各类自然灾害的监测和防治，维护人民生命财产安全。

（四）加强人口资源环境协调发展，促进人与自然和谐

扎实做好人口和计划生育工作，大力实施“少生快富”工程，进一步降低生育水平，有效治理出生人口性别比偏高等问题，提高出生人口素质。到2011年人口出生率、人口自然增长率分别控制在12‰和6‰以内。大力发展循环经济，推进资源节约、综合利用和清洁发展，逐步形成低投入、低消耗、低排放和高效率的节约型增长方式，促进资源节约型产业发展，积极开展节约型社会创建活动。继续实施好以退耕还林和封山育林为重点的生态林业工程，实施石漠化综合治理工程，力争森林面积达到543万亩以上，覆盖率达到40%以上，初步建成“两江”上游重要生态屏障。建立生态环境保护评价体系和补偿机制。加强城市环境综合治理，改善农村生活环境和村容村貌。加强污染专项整治，强化污染物排放总量控制，重点解决水、大气、土壤污染等危害人民群众身心健康的重大环境问题。

（五）加强民主法制和精神文明建设，培育文明道德新风尚

自觉接受人大及其常委会的法律监督和工作监督，认真向人大及其常委会报告工作；自觉接受政协的民主监督，认真向政协通报工作。认真听取各民主党派、工商联、无党派人士和各人民团体的意见，自觉接受新闻舆论和社会公众监督。发展基层民主，加强基层自治组织建设。进一步加大政务、村务、厂务公开力度。坚持和完善民族区域自治制度。依法管理宗教事务。抓好国防教育和动员工作，大力加强国防后备力量建设，增强全民国防意识，争创全国全省“双拥模范市”。支持工会、共青团、妇联、科协、残联、红十字会等人民团体依法按章开展工作。做好老干、档案、史志、外事、侨务和对台工作。做好“五五”普法工作，切实加强法律援助和法律服务工作。支持人民法院和人民检察院开展工作。

继续加强理想信念教育和社会主义思想道德建设，大力弘扬以爱国主义为核心的民族精神和以改革创新为核心的时代精神，树立“八荣八耻”社会主义荣辱观，建设社会主义核心价值体系。以提高公民道德素质为核心，在全社会倡导爱国守法、明礼诚信、团结友善、勤俭自强、敬业奉献的基本道德规范，使广大人民群众始终保持昂扬向上的精神状态。积极开展“热爱安顺、建设安顺”的主题教育活动，激发各族干部群众支持和投身安顺建设的热情。深入开展“整脏治乱”专项行动，改善全市城乡面貌。认真开展“四进社区”、“四在农家”活动，广泛开展和谐社区、和谐家庭和各种形式的群众性精神文明创建活动，移风易俗，积极引导城乡居民形成科学健康文明的生活方式，努力提高全社会的文明水平。

五、加强政府自身建设，提高构建新安顺行政能力

新一届政府肩负着承前启后、继往开来，带领全市人民奋力提速发展，实现黔中崛起，建设繁荣富裕秀美文明和谐新安顺的重任，必须进一步加强政府自身建设，在提高行政能力、狠抓落实上下功夫。

（一）转变政府职能，推进政府管理创新

按照转变职能、权责一致、强化服务、改进管理的要求，深化行政管理体制改革，大力推进政企分开、政事分开、管办分离，优化机构设置，更加重视履行社会管理和公共服务职能，创新公共服务体制，改进公共服务方式，逐步形成惠及全市群众的基本公共服务体系。深化行政审批制度改革，进一步减少和规范行政审批事项，简化办事程序，创新管理制度，降低行政成本。健全科学民主决策机制，完善重大决策的规则和程序，实行人民建议征集制度与群众利益密切相关的重大事项社会公示制度、社会听证制度、专家咨询制度，提高决策的透明度和公众参与度。加快电子政务建设，建成市、县、乡三级政务网络，逐步推行网上办公和网上审批，推进公共服务信息化，提高政府工作效率。

（二）改进工作作风，提高政府执行力

勤政为民，恪尽职守，以强烈的事业心和责任感，艰苦奋斗，埋头实干，始终把服务于人民放在首位，常怀为民之心、常思为民之策、常兴为民之举，以“民有所需，我有所为”的工作精神，为人民群众多办实事好事。健全一级抓一级、一级对一级负责，环环相扣、层次分明的责任体系，以求实、务实、落实的工作作风，切实解决工作部署不执行、虚执行或乱执行的问题，坚决杜绝推诿扯皮、推卸责任、形式主义等种种执行不力的现象，在全市范围内营造出坚决执行、主动执行的良好氛围，确保党委政府政令畅通。深化人事制度改革，大力加强政府自身队伍建设，强化宗旨意识、大局意识、服务意识和效率意识，靠严格的要求、严密的制度和严明的纪律，不断改进工作作风，改善政府形象，努力使各级政府成为人民群众满意的政府。

（三）推进依法行政，建设法治政府

自觉运用法律手段调节经济和管理社会事务，严格依照法定权限和程序行使权力、履行职责、接受监督，不断提高依法行政的能力和水平，使行政管理走上民主化、科学化、法制化的轨道。加强行政执法、法制教育等工作，进一步明确执法权限，减少执法层级，完善执法程序，提高执法水平。完善行政复议、行政赔偿和补偿等制度，加大行政综合执法改革力度，进一步推动和规范相对集中行政处罚权工作，坚决克服多头执法、重复执法、交叉执法等现象。加强对政府工作人员和执法人员的监督管理，对执法不公、越权执法、执法犯法、徇私枉法等行为严查严办。

（四）坚持廉洁从政，加强党风廉政建设

继续抓好中央《建立健全教育、制度、监督并重的惩治和预防腐败体系实施纲要》和省委《意见》、市委《实施方案》的落实，坚持不懈地加强廉政建设和反腐败斗争。坚决查办贪污腐败案件，重点查处利用职权谋取非法利益的案件，认真治理重点领域的商业贿赂，坚决纠正教育收费和医疗服务中的不正之风，认真解决土地征收征用、城镇房屋拆迁、移民搬迁、企业重组改制以及拖欠建设工程款和农民工工资等突出问题，自觉维护人民群众的合法权益，为民、务实、清廉。进一步健全党风廉政建设领导体制和工作机制，构建完善、管用、有效的廉政监督机制，做到用制度管权、按制度办事、靠制度管人。加大行政监察力度，提高行政效能，进一步完善招投标制、工程监理制、审计制和代建制。全力塑造清正廉洁的政府形象。

2007年的工作建议

各位代表，2007年是新一届政府履行职责的开局之年，我们将高起点、高标准地开展工作，为全面实现新一届政府工作目标奠定良好基础。全市经济社会发展的主要预期目标是：生产总值增长14%以上；全社会固定资产投资增长18%以上；财政总收入和地方财政收入增长15%以上；农民人均纯收入和城镇居民人均可支配收入实际增长8%；城镇登记失业率控制在4.6%以内；确保人口出生率和自然增长率分别控制在13.5‰、7‰以内；单位生产总值能耗比上年降低4%以上。继续办好人民群众关心的“实事”。

一是扎实抓好“三农”工作。社会主义新农村建设试点工作取得实效性进展。全年粮食总产量达到76万吨以上。畜牧业产值占农业总产值的比重提高1.5个百分点以上。净减少绝对贫困人口和低收入人口各6100人，实施127个贫困村“整村推进”，完成2000人易地扶贫搬迁。解决农村1.4万户、6.7万人饮水困难和饮水安全问题。完成2万口沼气池建设任务。组织实施贫困地区劳动力转移培训和“阳光工程”培训各8000人。

二是努力做大做强工业经济。全年工业增加值完成42.74亿元，同比增长14%。抓好天马虹山轴承公司入驻双阳机械工业园区的工作。抓好烟叶薄片和茄尼醇项目、安顺电厂烟气脱硫项目的实施。积极推进青年云雀轿车项目实施。抓好铝深加工项目的建设和生产。支持启动航空城建设。继续开展“三赛一会”活动。

三是促进旅游业和第三产业加快发展。继续加强旅游基础设施建设，加快黄果树新城建设。完成黄果树风景区两个镇政府搬迁工作，启动黄果树镇半边街搬迁工程。启动建设安顺市旅游信息服务工程。继续办好瀑布节、油菜花节和屯堡文化旅游节。接待游客800万人次以上，实现旅游收入50亿元左右。强化对服务业发展的政策支持和引导，加快安顺市《城市商业网点规划》的实施。

四是加快城市和基础设施重点项目建设。完成黄果树大街建设，加快推进中华东西路改造，启动二环路、北山路、武当路和安普路建设。进一步加强市政设施建设。支持各县抓好以县城为重点的基础设施建设，进一步改善县城面貌。力争开工建设普定—紫云—望谟新公路。完成200公里通乡油路和500公里通村公路建设。配合做好镇胜高速公路、黄织铁路和光照、董箐、马马崖电站的建设工作，进一步做好黔中水利枢纽和黄家湾水利枢纽工程前期工作。实施城市管道燃气工程。配合实施好省移动公司第二呼叫中心的建设。继续抓好退耕还林等重点林业工程建设，推进石漠化治理。

五是继续深化改革和扩大对外开放。深化安顺多种经济成分共生繁荣试验区改革，力争成功申报为国家综合配套改革试点。进一步加大对外宣传力度，加强与香港、日本等地的旅游和经贸合作。抓好招商引资签约项目的落实。完成市、县区属国有企业改制工作。实行政府收支分类体制改革。通过“市带县”模式全面开展与国家开发银行的第二轮合作。

六是加快社会事业发展。做好迎接国家“两基”督导检查工作，确保西秀区通过省“两基”复查和“普实”验收。实施80个农村薄弱学校改造工程。推进全市科技创新体系建设，启动实施“十一五”科技攻关计划。进一步加强基层卫生院、计生服务站（室）和体育设施建设。确保平坝、普定、镇宁、紫云、关岭、黄果树6个县区新型农村合作医疗试点参合率达80%。加快计生综合改革步伐，全面完成计生目标任务。完成省下达的20户以上（含50户）自然村“村村通”工程建设任务。积极推进有线电视网络整合和数字化整体转换工作。提高安顺人民广播电台覆盖率，增设旅游频率。力争城镇新增就业1万人，其中下岗失业人员再就业3000人（“4050”人员再就业300人）；组织劳务输出3万人以上。深入推进“平安安顺”创建活动，加强社会治安综合治理，依法严厉打击各种犯罪活动，切实维护社会稳定。努力实现安全生产“双降”目标。

各位代表，回顾过去，我们成绩可喜；展望未来，我们充满信心。让我们更加紧密地团结在以胡锦涛同志为总书记的党中央周围，高举邓小平理论和“三个代表”重要思想伟大旗帜，全面贯彻落实科学发展观，在市委的正确领导下，团结和依靠全市各族人民，奋力提速发展，实现黔中崛起，努力谱写建设繁荣富裕秀美文明和谐新安顺的壮丽篇章。我们坚信，我们的宏伟目标一定能够实现，黔中大地一定会更加锦绣辉煌！
